# Deaths in children in England from SARS-CoV-2 infection during the first 2 years of the pandemic: a cohort study

**DOI:** 10.1136/bmjopen-2024-092627

**Published:** 2025-02-05

**Authors:** David Odd, Sylvia Stoianova, Tom Williams, Anna Thursby-Pelham, Shamez N Ladhani, Godwin Oligbu, Peter Fleming, Karen Luyt

**Affiliations:** 1 Cardiff University, Cardiff, UK; 2 University of Bristol, Bristol, UK; 3 University Hospitals Bristol and Weston NHS Foundation Trust, Bristol, UK; 4 Immunisations and Vaccine-Preventable Diseases Division, UK Health Security Agency, London, UK; 5 St George’s University of London, London, UK; 6 Centre for Child and Adolescent Health, University of Bristol, Bristol, UK; 7 The National Institute for Health and Care Research Applied Research Collaboration West, Bristol, UK

**Keywords:** COVID-19, Mortality, Child

## Abstract

**Abstract:**

**Objective:**

The aim of this analysis was to describe the epidemiology, demographics and characteristics of children and young people (CYP) who died of SARS-CoV-2 infection in England during the first 2 years of the pandemic.

**Design:**

The cohort investigated in this study is all CYP, born alive at, or after, 22 weeks of gestation, who died before their 18th birthday between 1 February 2020 and 31 March 2022 in England. All cases were reviewed to identify if SARS-CoV-2 probably, or possibly, contributed to death. Mortality rates were calculated, assuming a Poisson distribution, for the whole population, and split by demographics and patient characteristics.

**Setting:**

England.

**Participants:**

6389 CYP deaths in England reported to the National Child Mortality Database (NCMD).

**Main outcome:**

Risk of death.

**Results:**

88 of the 6389 deaths of CYP were identified as deaths probably due to COVID-19. Thus, COVID-19 was responsible for 1.4% of all deaths of CYP in this 26-month period. Overall mortality rate due to COVID-19 in CYP was 3.59 (2.88–4.42) per 1 000 000 person years, being highest in the youngest (< 5 years; 4.68 (3.16–6.68)) and oldest (16/17 years; 4.83 (2.57–8.26)) CYP. Asian and Black CYP had higher mortality than those from white backgrounds (p<0.001), and mortality rate increased with increasing deprivation. The majority (61/77, 79.2) of CYP who died of COVID had a documented life limiting condition.

**Conclusions:**

Mortality rates were highest in less than 5 years old. Despite social changes, and shielding of vulnerable CYP, children with life-limiting (but not necessarily life-threatening) conditions, appeared to have the highest mortality rates, similar to that seen in adults with comparable underlying conditions. The risk of death in more deprived neighbourhoods and in those from Asian and Black ethnic backgrounds was increased, and this was not explained by their other demographic characteristics.

**STRENGTHS AND LIMITATIONS OF THIS STUDY**

- Based on statutory child death reviews.
- High level of data completeness.
- Detailed measures on COVID-related deaths.
- Limited precision due to small numbers of individual events.
- Denominators based on population estimates.

## Introduction

 While the COVID-19 pandemic probably caused over 14 million deaths worldwide,^1^ children and young people (CYP) may have a significantly lower risk of death due to COVID-19 than adults.^2 3^ However, it was associated with broad and profound changes in how people lived and worked to protect those with perceived increased vulnerabilities to the virus, including closing of schools, shops and recreational activity (‘lockdowns’), put in place to reduce the spread of COVID-19.^4 5^ However, even now, it is unclear how effective the public health mechanisms were to reduce mortality in the most vulnerable groups.^6^ Identifying CYP at highest risk of severe illness and death after SARS-CoV-2 infection therefore remains critical in order to learn from the COVID-19 pandemic and to balance policy in the future to avoid unwarranted impacts of pandemic lockdowns and restrictions.^4^

Our previous study identified 25 CYP who died of COVID-19 during the first pandemic year in England and that most had an underlying comorbidity, particularly neurodisability or life-limiting conditions.^2^ Differences in ethnicity and deprivation were also identified, but numbers were too small to draw definitive conclusions, and changes in virulence, case fatality rates and the development of vaccinations are likely to have impacted on the pattern, and at-risk groups, during the course of the pandemic. In particular, vaccination of pregnant women,^7^ and which underlying vulnerabilities may be the most important remains unclear.

In England (total population 56 million), all CYP who die before their 18th birthday are reviewed by one of the 56 local Child Death Overview Panels (CDOP).^8^ From 1 April 2019, the National Child Mortality Database (NCMD) has collated data on all deaths in CYP,^8^ with death notifications to the NCMD required by statute within 48 hours.

This work provides expanded analysis including the period across the first 2 years from the start of the COVID-19 pandemic to capture the total impact of COVID-19 on child mortality. Its aim was to describe the epidemiology, demographics and characteristics of CYP who died of SARS-CoV-2 infection in England during the first 2 years of the pandemic and the national lockdowns.

## Methods

The cohort investigated in this study is all CYP resident in England, born alive at, or after, 22 weeks of gestation, who died before their 18th birthday between 1 February 2020 and 31 March 2022 (26 months). In the initial notification to NCMD (within 48 hours of the death), the CDOPs report baseline characteristics of the child; from which the following data were derived:

- Sex of individual (female, male, other (including not known)).
- Ethnic group (Asian or Asian British, Black or Black British, mixed, other (Arab or any other ethnic group), unknown, White) (derived from self-reported health records).
- Age at death.
- From the child’s home postcode, a measure of deprivation (see below), the rural or urban nature of the area and the region of England where the child lived.
- Gestational age at birth.

Information on learning isability was identified from the formal, multi-agency review of the death where possible.

Underlying population profile was obtained from the Office for National Statistics (ONS) 2021 census data.^9^ Population denominators, stratified by age (years), and sex and ethnicity (coded as above) were derived at the level of the Middle Layer Super Output Areas (MSOA). Deprivation was derived from the index of multiple deprivation,^10^ calculated from a number of measures (eg, employment, crime) across the local area. The population was split into five quintiles with equal total populations, with a lower value suggesting greater deprivation. Region of England was also derived from the MSOA. For multivariable analysis, age data were obtained at the resolution of less than 5, 5–15 and 16 or 17 years old.

### Data collection and linkage

To ensure comprehensive identification of comorbidities, NCMD notified deaths were linked to National Health Service (NHS) Hospital Episodes Statistics (HES) admitted patient care data. All existing records going back to the birth of the child were obtained from HES. A validated list of International Classification of Diseases, Tenth Revision codes was used to identify CYP with chronic comorbidities^11^ and life-limiting conditions^12^ using all diagnosis codes from the child’s hospital activity.

### SARS-CoV-2 data

Deaths notified to NCMD were linked using NHS number by UK Health Security Agency (UKHSA) pillar 1 and pillar 2 testing data to identify all CYP who died with a positive COVID-19 test. Pillar 1 testing occurred in health and care settings, while pillar 2 testing occurred in the community; both started in March 2020. From April 2020, the national protocol for sudden unexpected deaths in CYP included postmortem testing for COVID-19. All CYP who died having had a positive SARS-CoV-2 test were initially included, regardless of the time interval between positive test and death. This difference from the definition used for reporting adult deaths aimed to ensure all potential cases were identified for review.

### Identifying children and young people (CYP who died of SARS-CoV-2

Information held in NCMD of all CYP who died having had a positive SARS-CoV-2 test were reviewed to identify if SARS-CoV-2 clearly, probably, possibly or unlikely contributed to death.

Each case underwent review by two of three independent senior clinical experts in relevant fields (infectious disease, general paediatrics, paediatric intensive care) who classified each case using information recorded within NCMD when the assessment took place (April 2023). Each senior clinical expert was blinded to the opinion of the other reviewer. Where there was disagreement between the initial two experts, the third expert reviewed the information, and the classification with the majority was used. Deaths classified as clearly, probably or possibly due to COVID were included as CYP who died of COVID-19 (‘Likely COVID deaths’). Deaths where one of the experts classified that COVID-19 clearly, probably or possibly contributed but where there was an overall majority opinion that this was unlikely were used as a wider definition (‘possible COVID deaths’) and used only in a sensitivity analysis. The 61 deaths identified in our previous work were re-reviewed to ensure that the methodology was consistent across the whole cohort presented here and to ensure all latest clinical information was reviewed. With additional information now available, five deaths, previously classified as not COVID-19 related, were reclassified as likely COVID deaths, and one death, classified previously as a likely COVID death, reclassified as a Possible COVID death.

### Vaccination status

Deaths notified to NCMD were linked using NHS number by UKHSA to COVID-19 vaccination status from records held at UKHSA. Maternal vaccination data was returned on 25 May 2022 for children under 1 year who were born after 1 January 2021, alongside vaccination data of CYP aged 5 and over who died after 1 March 2021. A child was considered to be vaccinated if they had received two doses of a COVID-19 vaccine (all immunisation regimes in the UK used two-dose vaccines) and completed at least 2 weeks before the child’s death.^13^ A mother was considered to be vaccinated if she had two doses at least 2 weeks before the child’s birth.

### Statistical analysis

Initially, the number and characteristics of CYP who did not die of COVID-19 and those that did were compared. Differences between groups were compared using χ^2^. For presentation, the CYP who had tested positive for COVID-19, but died of another cause, were also identified, but all statistical tests throughout the work compared those thought to die of COVID-19, with all who did not.

Next, the mortality rate with 95% CIs was calculated, assuming a Poisson distribution. The population at risk (denominators) were derived from the 2021 UK census data (ONS)^9^ and allowed estimates of the population across England by age (in years), sex and ethnicity, down to the level of the MSOA, a geographic area containing around 8000 people. An unadjusted rate ratio (RR) was derived to compare mortality rate between patient characteristics, as well as a multivariable model to adjust for the other demographics available (coded as above). Due to limits in the data granularity, age was categorised as less than 5, 5–15 and 16 or 17 years old for the multivariable analyses.

Using the HES data, a number of deaths across the whole cohort, with and without life-limiting conditions (LLCs), were identified (onlinesupplemental etables 1  2). The relative odds of CYP with LLC dying of COVID-19, compared with those deaths without LLC, was derived for increasing number of LLCs and repeated for each category of LLC using a logistic regression model. Due to the variation in diagnosis by age, and the likely patterns of COVID-19 infection rates, all models used a random-effect multi-level model by age (in years) as a grouping variable. This model was repeated containing, and adjusting for, all other LLCs. Estimated population of children with LLCs was identified from previous work, and mortality rates of dying of COVID-19 for CYP with 0, 1, 2 or 3+ LLCs were estimated.^12^ This analysis was repeated for a number of specific diseases. As above, using the predicted populations of CYP with seven specific diseases reported in our previous work,^2^ as well as for CYP with cystic fibrosis, chronic renal failure, cerebral palsy and preterm birth (online supplemental etable 3), we derived an estimated rate of dying of COVID-19 for that patient population. The estimated populations at risk for the 11 specific conditions were identified from the existing literature.^212 14^,^24^

Two sensitivity analyses were performed. First, analysis was repeated using the wider category, where COVID-19 only possibly contributed to the death (see above). Second, the profile of LLCs and chronic conditions analysis was repeated including all CYP, and assuming that those without a linkage to HES data had no previous conditions.

Data are presented as median number (%), mortality rate (95% CI) per 1 000 000 CYP per year, OR (95% CI) or RR (95% CI). All tests were two-sided, unless otherwise stated, and analysis was performed using Stata V.17.

### Patient and public involvement

Parent, patient and public involvement (PPPI) guided the design and setting up of the NCMD at establishment and real-time child mortality surveillance system at the beginning of the COVID-19, but were not involved in the development of the specific research question, or the design of this specific analysis. Results of this work will be disseminated to our partners through the ongoing programme of PPPI work.

### Ethics approval and consent to participate

The NCMD legal basis to collect confidential and personal level data under the Common Law Duty of Confidentiality has been established through the Children Act and associated Child Death Review Statutory & Operational Guidance. The NCMD legal basis to collect personal data under the General Data Protection Regulation (GDPR) without consent is defined by GDPR Article 6 (e) Public task and 9 (h) Health or social care (with a basis in law), and consequently the need for consent was waived by the ethics committee (Central Bristol NHS Research Ethics Committee).

## Results

**Figure 1:**
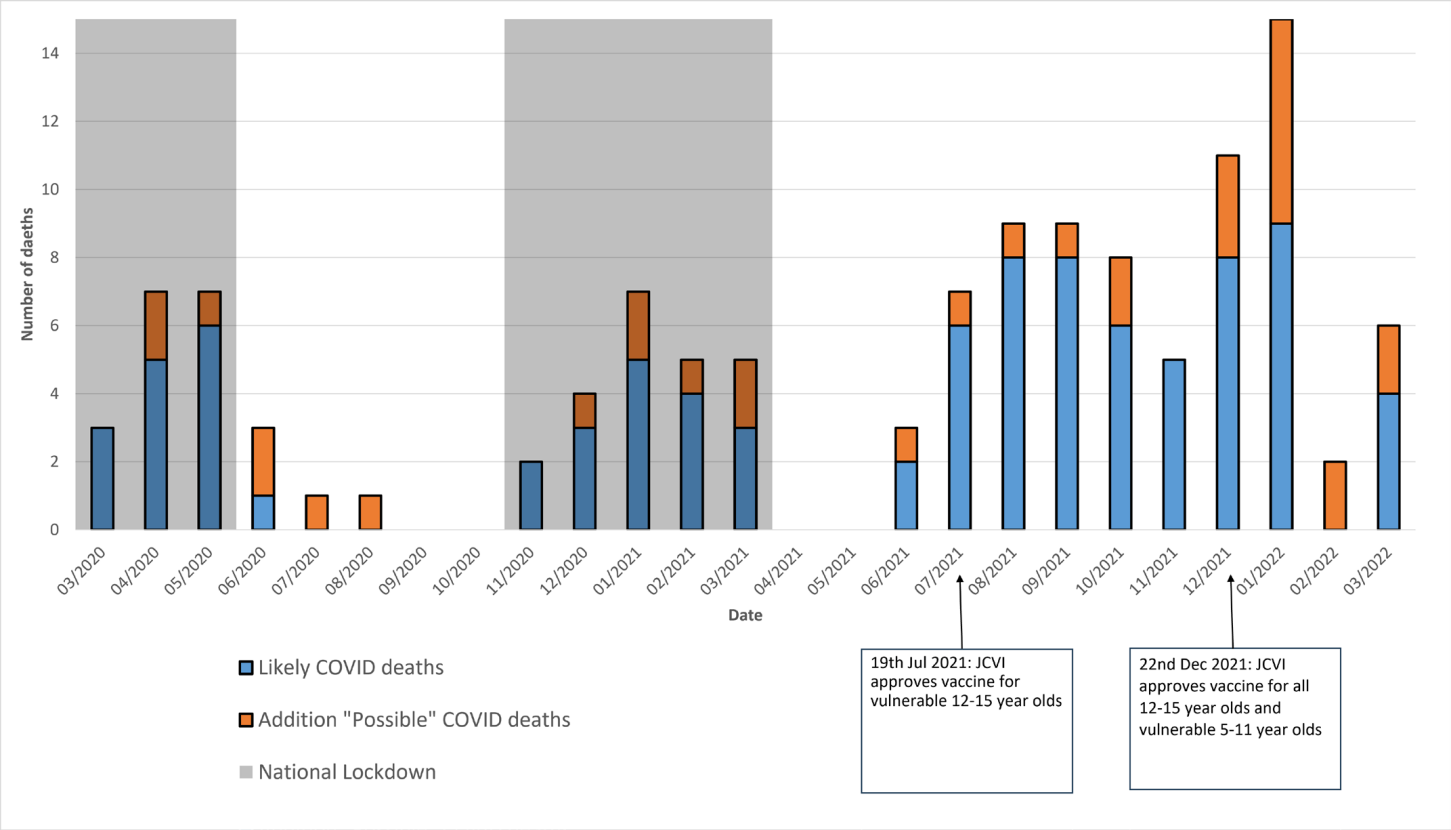
Number of COVID-19 deaths in children and young people by calendar month and year in England. JCVI, Joint Committee on Vaccination and Immunisation.

There were 6389 deaths of CYP reported to the NCMD over the 26-month surveillance period (online supplemental etable 4). Figure 1 summarises the number of childhood deaths likely due to COVID-19, over the 26-month surveillance period, for both the main measure (likely COVID deaths) and the sensitivity measure (possible COVID deaths) (see above). Individual events are rare, making interpretation difficult, but peaks of death at the start of the pandemic, across the first winter and then a gradual rise after cessation of National Lockdowns in Spring 2021 appear apparent.

Of these, 346 were positive for COVID-19 before or around the time of their death, and 88 (1.4%) were identified as deaths likely due to COVID-19; in the remaining 258 cases (4.0%) that had a record of a positive COVID-19 test, the virus was not assessed to be responsible for the death. Overall, 25.4% of deaths, where there was a positive COVID-19 test, were assessed as likely caused by COVID-19, but this appeared to change over time, 53.6% (15/28) before August 2020 and 23.0% (73/318) for the rest of the period. Of the 88 COVID-19 deaths, 17 (19.3%) had their first positive COVID-19 test on the day of, or after, their death, including nine CYP who initially presented as sudden unexpected deaths in infancy or childhood.

The profile of COVID-19 deaths varied by age (p<0.001), with the highest proportion of deaths attributed to COVID-19 in the 10–14-year-old category (n=21 (3.5%)) (online supplemental etable 4). The profile of COVID-19 deaths also varied by ethnicity (p<0.001), deprivation (p=0.01), but not by sex (p=0.30) or region of England (p=0.75). Of all the 6389 CYP who died, only 84 had been fully vaccinated (two doses more than 14 days before their death), and none of these were thought to have died of COVID-19, although (3.6%) of them had a positive COVID-19 test within 28 days of their death.

Among mothers of infants who died, 281 had been fully immunised, and no COVID-19-related deaths of infants were identified in infants of immunised mothers, although one (1.4%) child had a positive COVID-19 test within 28 days of their death. The majority of COVID-19 deaths occurred in the hospital (72/88 (81.8%)), 10 at home, two at a hospice and four in other locations. Learning disability data were available on 1548/1828 deaths in CYP aged 5–17 years. CYP with learning disabilities who died were twice as likely (30/550 (5.5%)) to have died of COVID-19 than those without learning disabilities (22/998 (2.2%)) (p=0.001).

### Mortality rates due to COVID-19

**Figure 2:**
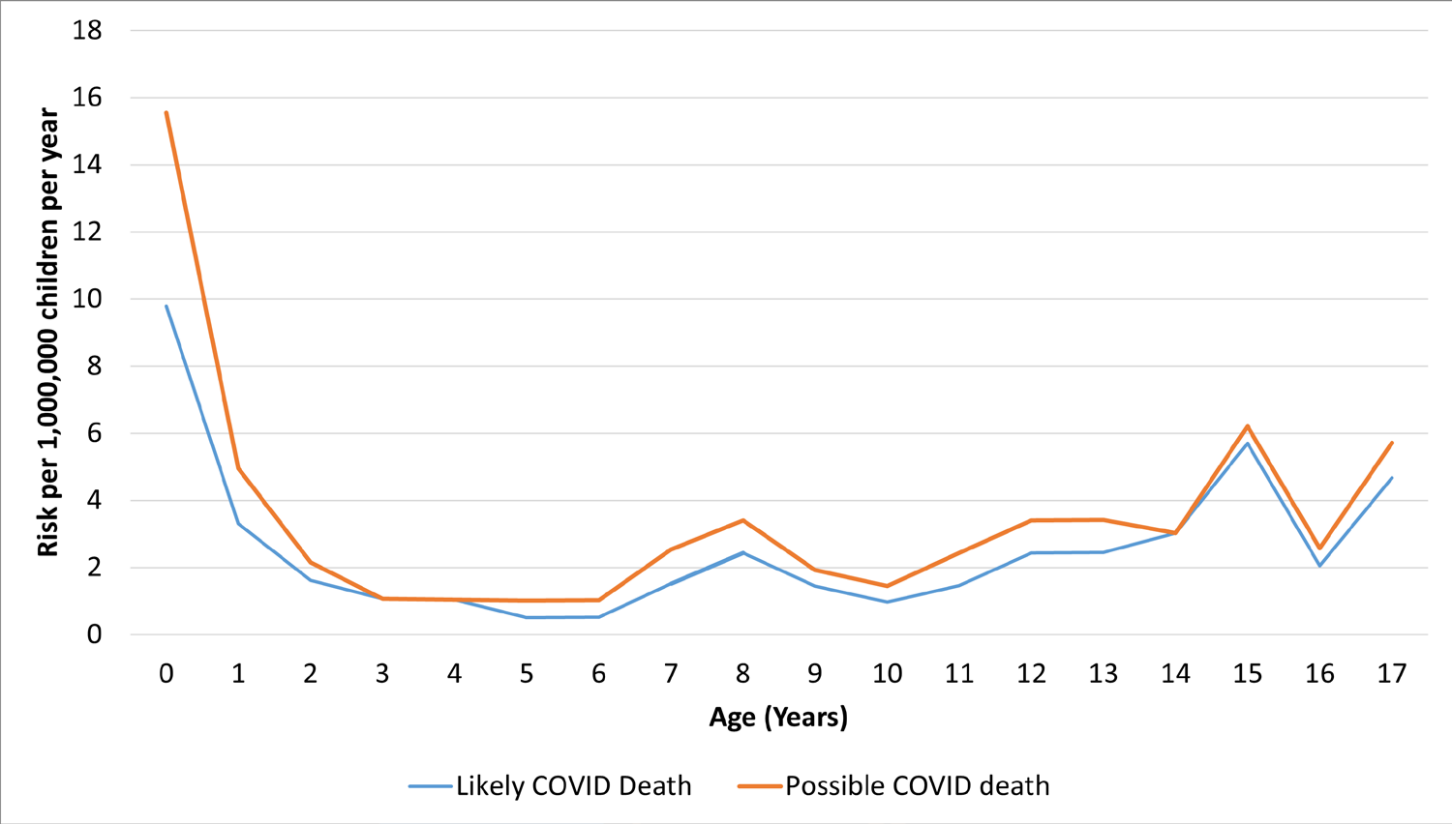
Rate of death of likely, and possible, COVID-19 per 1 000 000 children per year by age at death (in years).

The overall mortality rate due to COVID-19 in CYP was estimated to be 3.59 (2.88–4.42) per 1 000 000 person years, being higher in the youngest (<5 years; 4.68 (3.16–6.68) per 1 000 000 person years) and oldest (16/17 years; 4.83 (2.57–8.26) per 1 000 000 person years) CYP age categories (table 1). CYP aged 5–15 years had the lowest overall rate (2.92 (2.13–3.90) per 1 000 000 person years), and this persisted in the adjusted Poisson regression model (RR 0.58 (0.36–0.92), p=0.02). Figure 2 shows the rate of death by age by individual years for both the main measure (likely COVID deaths) and the sensitivity measure (possible COVID deaths) (see above). There was no difference in the COVID-19 mortality rate by sex (unadjusted, p=0.98; adjusted, p=0.92).

**Table 1:** COVID-19 mortality rates over 26 months, split by child characteristics

Characteristic	N	Died of COVID-19 (n=88)		Rate ratio (RR)			
		Rate/1 000 000 person years (95% CI)	P	Unadjusted	P	Adjusted*	P
				RR (95% CI)		RR (95% CI)	
All deaths	88	3.59 (2.88 to 4.42)	NA	NA	NA	NA	NA
Age			0.0796				
<5	30	4.68 (3.16 to 6.68)		1 (Ref)	NA	1 (Ref)	NA
5–15	45	2.92 (2.13 to 3.90)		0.62 (0.39 to 0.99)	0.045	0.58 (0.36 to 0.92)	0.02
16/17	13	4.83 (2.57 to 8.26)		1.03 (054 to 1.98)	0.923	0.96 (0.50 to 1.84)	0.90
Sex			0.9831				
Female	43	3.60 (2.60 to 4.84)		1 (Ref)	NA	1 (Ref)	NA
Male	45	3.58 (2.61 to 4.79)		1.00 (0.66 to 1.51)	0.983	1.02 (0.67 to 1.56)	0.92
Ethnicity†			<0.001				
Asian or Asian British	28	9.27 (6.16 to 12.40)		4.02 (2.49 to 6.51)	<0.001	6.88 (4.14 to 11.42)	<0.001
Black or Black British	11	7.86 (3.92 to 14.07)		3.41 (1.75 to 6.64)	<0.001	8.05 (3.95 to 16.43)	<0.001
Mixed	5	3.00 (0.97 to 6.99)		1.30 (0.51 to 3.29)	0.580	1.60 (0.63 to 4.07)	0.32
Other‡	2	3.07 (0.37 to 11.09)		1.33 (0.32 to 5.51)	0.692	2.24 (0.53 to 9.37)	0.27
White	41	2.30 (1.65 to 3.13)		1 (Ref)	NA	1 (Ref)	NA
Deprivation			<0.001				
1/2	36	7.95 (5.57 to 11.00)		5.93 (2.76 to 12.75)	<0.001	15.20 (6.75 to 34.21)	<0.001
3/4	15	3.36 (1.88 to 5.55)		2.51 (1.06 to 5.92)	0.036	6.06 (2.49 to 14.75)	<0.001
5/6	24	5.27 (3.38 to 7.84)		3.93 (1.77 to 8.75)	<0.001	8.24 (3.61 to 18.82)	<0.001
7/8	5	1.00 (0.32 to 2.33)		0.74 (0.24 to 2.27)	0.603	1.15 (0.37 to 3.54)	0.81
9/10	8	1.34 (0.58 to 2.64)		1 (Ref)	NA	1 (Ref)	NA
Region			0.6464				
East Midlands	5	2.42 (0.79 to 5.65)		1 (Ref)	NA	1 (Ref)	NA
East of England	8	2.89 (1.25 to 5.69)		1.19 (0.39 to 3.64)	0.758	0.98 (0.32 to 3.00)	0.97
London	18	4.55 (2.70 to 7.19)		1.88 (0.70 to 5.06)	0.212	1.38 (0.50 to 3.79)	0.53
North East	3	2.74 (0.57 to 8.01)		1.13 (0.27 to 4.74)	0.865	1.98 (0.47 to 8.32)	0.35
North West	14	4.30 (2.35 to 7.21)		1.77 (0.64 to 4.93)	0.271	2.30 (0.82 to 6.47)	0.12
South East	14	3.22 (1.71 to 5.51)		1.33 (0.47 to 3.73)	0.589	0.98 (0.35 to 2.73)	0.96
South West	7	3.09 (1.24 to 6.36)		1.28 (0.40 to 4.02)	0.678	1.41 (0.45 to 4.47)	0.56
West Midlands	7	2.60 (1.04 to 5.35)		1.07 (0.34 to 3.38)	0.905	1.22 (0.39 to 3.87)	0.73
Yorkshire and Humber	13	5.44 (2.90 to 9.30)		2.24 (0.80 to 6.30)	0.124	3.10 (1.10 to 8.73)	0.03

* Adjusted for all other covariates listed in the table.

† One infant had missing data for ethnicity.

‡ Arab and any other ethnicity.

After adjusting for other covariates, COVID-19 deaths remained strongly associated with ethnicity (p<0.001) and deprivation (p<0.001). The highest mortality rate by ethnicity was among CYP from an Asian or Asian British background (9.27 (6.16–12.40) per 1 000 000 person years), followed by Black or Black British backgrounds (7.86 (3.92–14.07) per 1 000 000 person years). CYP from White backgrounds had the lowest rate (2.30 (1.65–3.13) per 1 000 000 person years). This difference persisted in the adjusted model, with Asian or Asian British (RR 6.88 (4.14–11.42), p<0.001) and Black or Black British (RR 8.05 (3.95–16.43), p<0.001) having a higher risk than White children.

The highest COVID-19 mortality risk by deprivation was among CYP in the lowest (most deprived) quintile (7.95 (5.57–11.00) per 1 000 000 person years). The COVID-19 mortality rate reduced with decreasing levels of deprivation, with those in the most deprived quintile having a RR of 15.20 (6.75–34.21) (p<0.001) when compared with those in the least deprived category. COVID-19 mortality rates were similar across the English regions (p=0.65).

### Life-limiting and chronic conditions

CYP deaths were successfully linked to HES for 5444/6389 (85.2%) of the whole cohort and 77/88 (87.5%) of those who died of COVID-19: the subsequent analyses are based on this cohort. CYP with a greater number of LLCs had a higher risk of dying of COVID-19 (OR 1.18 (1.03–1.34), p=0.02) (table 2) (online supplemental etable 5). The majority (61/77, 79.2%) of CYP who died of COVID had a documented LLC. In the age-adjusted analysis, respiratory (OR 4.60 (2.84–7.47), p<0.001), circulatory (OR 2.56 (1.20–5.48), p=0.02) and genitourinary problems (OR 2.13 (1.29–3.53), p=0.003) were independently associated with death due to COVID-19, while neoplasms (OR 0.15 (0.05–0.50), p=0.002) and haematological illness (OR 0.35 (0.14.0.88), p=0.03) were associated with a lower risk. In the multivariable logistic regression model, the higher incidence of deaths in CYP with respiratory (OR 5.08 (2.86–9.02), p<0.001) and genitourinary LLCs (OR 1.95 (1.09–3.50), p=0.03) and the lower incidence in those with neoplasms (OR 0.21 (0.06–0.82), p=0.03) persisted. While most CYP who died of COVID-19 (69; 88.3%) had at least one known chronic disease, there was only weak evidence for an increasing number of chronic conditions being associated with a higher risk of dying of COVID-19 (OR 1.05 (1.00–1.11; p=0.07).

**Table 2:** Life-limiting and chronic conditions in children who died, number of children and relative odds after adjusting for age, split by death from COVID-19 (n=5444)

Characteristic		Died of other causes		Died of COVID-19		Adjusted for age	P*	Adjusted for age and other LLC/chronic conditions	P†
		N	%	N	%	OR (95% CI)*		OR (95% CI)†	
All Deaths		5367	98.6	77	0.9	NA	NA		
Number of LLCs	0	1925	99.2	16	0.8	1.18 (1.03 to 1.34)	0.02	NA	NA
	1	1505	99.1	14	0.9			NA	NA
	2	906	98.2	17	1.8			NA	NA
	3+	1031	97.2	30	2.8			NA	NA
Number of chronic conditions	0	1235	99.3	9	0.7	1.05 (1.00 to 1.11)	0.07		
	1	665	99.6	3	0.5				
	2	727	99.2	6	0.8				
	3+	2740	97.9	59	2.1				
LLC categories									
Neurological		1071	96.9	34	3.1	1.65 (1.00 to 2.74)	0.05	0.80 (0.43 to 1.49)	0.47
Haematology		450	98.9	5	1.1	0.35 (0.14.0.88)	0.03	0.53 (0.18 to 1.59)	0.26
Oncology		532	99.4	3	0.6	0.15 (0.05 to 0.50)	0.002	0.21 (0.06 to 0.82)	0.03
Metabolic		263	97.1	8	3.0	1.57 (0.73 to 3.35)	0.25	1.18 (0.53 to 2.65)	0.68
Respiratory		518	94.0	33	6.0	4.60 (2.84 to 7.47)	<0.001	5.08 (2.86 to 9.02)	<0.001
Circulatory		182	95.8	8	4.2	2.56 (1.20 to 5.48)	0.02	1.70 (0.75 to 3.86)	0.20
Gastrointestinal		268	97.5	7	2.6	1.54 (0.69 to 3.42)	0.29	0.79 (0.33 to 1.90)	0.60
Genitourinary		689	96.6	24	3.4	2.13 (1.29 to 3.53)	0.003	1.95 (1.09 to 3.50)	0.03
Perinatal		1406	99.1	13	0.9	1.47 (0.75 to 2.89)	0.26	0.87 (0.44 to 1.69)	0.67
Congenital		1191	98.6	17	1.4	1.18 (0.68 to 2.05)	0.56	0.69 (0.38 to 1.24)	0.22
Other		671	98.4	11	1.6	0.75 (0.39 to 1.45)	0.34	0.77 (0.38 to 1.57)	0.47

* Comparison of odds seen between CYP likely dying of COVID-19 with condition and all other children and young people adjusted for age at death .

† Comparison of odds seen between CYP likely dying of COVID-19 with condition and all other children and young people adjusted for age at death and other LLCs.

CYPchildren and young peopleLLClife-limiting condition

Using published childhood population estimates, the COVID-19 mortality rate (per 1 000 000 person-years) was 0.66 (0.38–1.07) for CYP without a LLC, 106.37 (58.15–178.46) for those with one, 449.15 (261.65–719.14) for two and 1646.81 (1111.10–2350.93) for three or more LLCs (table 3) (online supplemental etable 6). The specific condition with the highest estimated mortality rate was life-limiting neurodisability (1072.89 (743.01–1499.26) per 1 000 000 person-years). Of note, all CYP with epilepsy or asthma, who died of COVID-19, had another underlying LLC, and no child with diabetes mellitus died of COVID-19 during the study period.

**Table 3:** Estimated COVID-19 mortality rates by life-limiting, chronic and specific conditions (n=5444)

Characteristic		Died of other causes		Died of COVID-19		COVID-19 mortality rate per 1 000 000 person years	Estimated population at risk
		N	%	N	%		
All deaths		5367	98.6	77	0.9	3.59 (2.88–4.42)	
Number of LLC conditions	0	1925	99.2	16	0.8	0.66 (0.38–1.07)	24 379 096
	1	1505	99.1	14	0.9	106.37 (58.15–178.46)	131 621
	2	906	98.2	17	1.8	449.15 (261.65–719.14)	37 849
	3+	1031	97.2	30	2.8	1646.81 (1111.10–2350.93)	18 217
Specific additional conditions							
Asthma*		175	97.2	5	2.8	2.50 (0.81–5.83)	2 000 027
Diabetes mellitus*		40	100.0	0	0.0	0.00 (0.00–56.37)†	65 455
Epilepsy		808	96.8	27	3.2	234.22 (154.36–340.78)	115 274
Sickle cell disease		3	75.0	1	25.0	111.10 (2.81–619.00)	9001
Trisomy 21		67	91.8	6	8.2	280.61 (102.98–610.77)	21 382
Oncology		532	99.4	3	0.6	88.68 (18.28–259.17)	33 828
Cardiology (congenital)		828	97.8	19	2.2	97.44 (58.66–152.16)	195 000
Life-limiting neurodisability		1071	96.9	34	3.1	1072.89 (743.01–1499.26)	31 690
Cystic fibrosis		6	75.0	2	25.0	256.54 (31.07–926.72)	7796
Chronic renal failure		58	96.7	2	3.3	948.32 (114.85–3425.65)	2109
Cerebral palsy		372	95.6	17	4.4	284.85 (165.94–456.08)	59 680
Preterm birth		2439	99.1	22	0.9	12.17 (7.63–18.42)	1 808 065

* All child who died of Aasthma or Ddiabetes Mmellitus had at least 1one additional LLC.

† OR for Ddiabetes Mmellitus is derived one-sided with 97.5% confidence intervalsCIs due to no children in the COVID-19 group.

LLClife-limiting condition

When repeating the analysis using the lower level of proof (‘possible COVID deaths”), 120/6389 (1.9%) of all deaths were identified as possibly caused by COVID-19, while 226/6389 (3.5%) were positive for the virus around the time of death, but not considered likely to have died of COVID-19. The estimated mortality rate for CYP dying of SARS-CoV-2, increased to 4.89 (4.06–5.85) per 1 000 000 person years. In this cohort, 102/120 (85.0%) of the possible COVID-19 deaths were linked to a HES record, and, of these, a total of 83/102 (81.4%) had evidence of a LLC, and 61/102 (59.8%) had a chronic condition.

Repeating the analysis with all CYP, and assuming that those without a linkage to the HES data had no LLC or chronic condition, resulted in similar findings to the main analysis, but with 61/88 (69.3%) having an LLC and 68/88 (77.3%) having evidence of a chronic disease.

Due to the unexpected high number (n=17) of infant deaths (age <1 year), a more detailed post-hoc assessment was undertaken. The median age at death of these infants was 9 (IQR 0–45) days, 12 (70.6%) were male, nine (52.9%) were of white ethnicity, nine (52.9%) were born preterm (less than 37 weeks gestation) and 12 (70.6%) died in the second year of the pandemic. Six had congenital heart disease, and all deteriorated rapidly after SARS-CoV-2 infection. Six of the infants were born to a mother with active COVID-19 disease during labour; these were all born prematurely and died in the first 28 days of life. Four other infants presented with sudden unexpected death in infancy, which later was identified as likely due to COVID.

## Discussion

We estimate that in England, 88 CYP died of COVID-19 during the first 26 months of the COVID-19 pandemic, with an estimated mortality rate of 3.59 (2.88–4.42) per 1 000 000 person years. At the beginning of the pandemic, most CYP who were found to be SARS-CoV-2 positive around the time of their death were thought likely to have died of the virus itself. This proportion reduced during 2020, but rose in the first months of 2021, at the time of the Omicron outbreak. The largest number of deaths from COVID-19 in CYP occurred in the youngest (under 1 year) and oldest (over 10 years). This translated to a highest predicted risk of death from SARS-CoV-2 in the youngest and oldest CYP. The risk of dying of COVID-19 was also highly patterned by ethnicity and deprivation, substantially higher for those CYP from Asian or Black backgrounds, or in the more deprived areas. We identified a number of underlying conditions that were associated with a higher mortality risk due to COVID-19. Children with neurodisability, but also sickle cell disease, Trisomy 21, congenital cardiac disease and cystic fibrosis were all likely to have a much higher chance of SARS-CoV-2 being considered a cause of death compared with the overall population. Although, as in our previous work, all children with epilepsy or asthma who likely died of COVID-19 also had an LLC. Interestingly, children dying with an oncological condition had a lower proportion of deaths from COVID-19 (0.6%) than the rest of the population (0.9%), but still had an estimated higher chance of death from COVID-19 (88.68 (18.28–259.17) per 1 000 000 person years) than the general population (3.59 (2.88–4.42) per 1 000 000 person years), likely mediated by the much higher overall mortality in this group (533 deaths from an estimate population of 33 828 children).

Indeed, overall, 80% of CYP who died of COVID-19 had an LLC, and 90% had an underlying chronic condition. We identified no deaths in vaccinated CYP, perhaps for two reasons: a delay in vaccine availability for children, because of prioritisation of adults (although vaccine was recommended for neurodisabled older children soon after it was licensed in the UK (January 2021)), as well as low uptake of vaccine in this proposed high risk group early in the pandemic.^25^ There were also no deaths in infants born to vaccinated mothers during the first 2 years of the pandemic, although again, effective take-up was only seen towards the end of the study periods.^26^

### Interpretation

It is important to distinguish between the wider impact of the COVID-19 pandemic and the direct infectious burden of COVID-19 in CYP. In England, child mortality dropped to its lowest level ever recorded during the first year of the pandemic, with reductions in deaths from other infectious agents, substance abuse and from underlying, chronic disease, reducing substantially during the main period of the pandemic, and the social lockdowns around it.^4^,^6^ The 88 deaths identified in this analysis occurred despite this overall reduction in mortality, and despite the social distancing and other public health initiatives implemented while the vaccination roll-out was realised. Furthermore, while others have reported lower levels of severe disease in CYP during the omicron variant era,^3^ in this work, we saw a rise in the number of deaths during this time, most likely due to a higher number of infections. However, infection rates, patterned by underlying vulnerabilities, are complex and interpretation consequently limited.

Data from the USA estimated a crude death rate of 1.0 per 100 000 population for individuals aged 0 to 19 years during a similar surveillance period, around three times higher than that seen in England (0.36 per 100,000). Indeed, in the USA, COVID-19 mortality between August 2021 and July 2022 was 2% and the eighth most common cause of death in this age group^27^ (compared with 1.4% in this work). Some of the difference may be explained by the wider age range reported in that work (0–19 years)^27^ and the limited data available on the US deaths compared with the NCMD dataset.

Consistent with other work,^27^ the highest risk of CYP COVID-19 deaths were in infants and after the 10th year of life. The higher mortality rate seen here in infants may have been previously unrecognised due to the substantial mortality already seen in this age range^24^ although it may reflect higher infection rate. However, maternal vaccination is likely to reduce stillbirth, preterm births and neonatal intensive care unit admission, in addition to reductions in COVID-19 infection during the first 6 months of life.^7 28^

The association of COVID-19 deaths with underlying conditions in CYP (especially severe neurodisability) is not unexpected and consistent with other work.^29^ Around 10% of COVID deaths had no evidence of any long-term disease, including common childhood ailments, such as asthma, and is similar to the proportion of COVID-19 deaths across all ages, including adults, where no pre-existing condition was identified (England and Wales data, 11.8%).^30^ However, for CYP with underlying conditions, it can be difficult to assess the contribution of the virus infection to death, compared with the contribution of the underlying condition itself. For some groups (eg, malignancies and neonatal deaths), overall mortality has not measurably changed over the 3 years of the pandemic^6^ as, even if, the individual additional risk of COVID in these groups may be substantial, it remains difficult to identify this in settings where the base-line mortality is already very high. In contrast, the social changes around the pandemic reduced the circulation of other infections, and without COVID-19, many of these CYP may have succumbed to a different pathogen. Currently, with all mitigations removed, other viruses are circulating, and it is essential we continue to assess the ongoing contribution of this virus to severe disease, and deaths, in CYP. Finally, of course, all the mortality rates, and RRs, reported here were after lockdown implementations and personal shielding of these vulnerable groups. While social distancing, and other interventions, may have reduced some of the health inequalities in England,^4^ the stark associations with ethnicity and deprivation are consistent with ONS data in adults^30^ and appear disproportionate to the already recognised inequalities in child mortality^31^ possibly offsetting any possible progress seen over the last decade.

### Limitations

This work is based on statutory data reported to NCMD, and previous work has shown good validation and coverage.^32^ However, there were some missing demographic data (eg, ethnicity), and some linkage with routine data may have been insufficient; and this should be considered when interpreting our findings. We could only identify a HES record in 83% of CYP. For the main analysis, we excluded those where a record was not obtained. If these records were not present (possibly due to the child not previously having accessed in-patient healthcare), then the proportion of those CYP dying of COVID-19, without an underlying LLC or chronic conditions, appears to double.

The coding of cause of death (ie, COVID-19 or not) has potential limitations, but we used a similar process to previous work,^2^ and we were able to access a broad range of data to assess the contribution of COVID-19 to the death in each case. We also used routine HES records (a routine administrative database) to identify underlying conditions, and miscoding in some of these records is possible. In addition, the estimates of underlying conditions used here, especially for uncommon conditions, may not be accurate, and therefore, the relative mortality rates between different conditions needs to be interpreted with caution. The main limitation of this work, despite national coverage over the first 26 months of the COVID-19 pandemic, is the very small number of COVID-19 fatalities in CYP, which inevitably limits interpretation of subgroup analyses and precision of our estimates. In addition, we also did not have infection status data across the whole population (where it was known), so mortality rates presented here are population rates, rather than case-specific fatality rates, and consequently differences between groups may represent differential exposure rather than increased vulnerability.

### Conclusion

COVID-19 was responsible for 1.4% of all deaths of CYP in England between 2020 and 2022, but the proportion ranged from 0.4% in infants to 3.2% in CYP between 15 and 17 years. However, mortality rates were highest in infants. Despite social changes, and shielding of vulnerable CYP, children with underlying and life-limiting (but not necessarily life-threatening) conditions appeared to have the highest mortality rates, similar to that seen in adults with comparable underlying conditions. The risk of death in England’s more deprived neighbourhoods and in children from Asian and Black ethnic backgrounds appears substantially higher than their peers and was not explained by their other demographic characteristics. The number of children, or mothers, vaccinated during this period was too low to adequately assess its impact; but no vaccinated child, or infant with a vaccinated mother, died of COVID-19 in this work.

## supplementary material

10.1136/bmjopen-2024-092627 online supplemental file 1

10.1136/bmjopen-2024-092627 online supplemental file 2

10.1136/bmjopen-2024-092627 online supplemental file 3

10.1136/bmjopen-2024-092627 online supplemental file 4

10.1136/bmjopen-2024-092627 online supplemental file 5

10.1136/bmjopen-2024-092627 online supplemental file 6

## Data Availability

Data are available upon reasonable request.
