# Supplementary material for: Deaths in children in England from SARS-CoV-2 infection during the first 2 years of the pandemic: a cohort study
Source: BMJ Open. 2025 Feb 5;15(2):e092627. doi: 10.1136/bmjopen-2024-092627 (PMC11800287; doi:10.1136/bmjopen-2024-092627)
Supplement: online supplemental file 1 [file bmjopen-15-2-s001.docx]

**eTable 1. Life-limiting condition ICD-10 codes**

| **All** | **ICD-10 Description** | **Category** |
| --- | --- | --- |
| A17 | Tuberculosis of nervous system | Neurology |
| A810 | Creutzfeldt-Jakob disease | Neurology |
| A811 | Subacute sclerosing panencephalitis | Neurology |
| B20-B24 | Human immunodeficiency virus [HIV] disease | Haematology |
| C00-C97 | Malignant neoplasms | Oncology |
| D33 | Benign neoplasm of brain and other parts of central nervous system | Oncology |
| D43 | Neoplasm of uncertain or unknown behaviour of brain and central nervous system | Oncology |
| D444 | Neoplasm of uncertain behavior of craniopharyngeal duct | Oncology |
| D48 | Neoplasm of uncertain or unknown behaviour of other and unspecified sites | Oncology |
| D561 | Beta thalassemia | Haematology |
| D610 | Constitutional aplastic anaemia | Haematology |
| D619 | Aplastic anemia, unspecified | Haematology |
| D70 | Agranulocytosis | Haematology |
| D761 | Hemophagocytic lymphohistiocytosis | Haematology |
| D81 | Combined immunodeficiencies | Haematology |
| D821 | Di George's syndrome | Haematology |
| D83 | Common variable immunodeficiency | Haematology |
| D891 | Cryoglobulinemia | Haematology |
| E310 | Autoimmune polyglandular failure | Metabolic |
| E348 | Other specified endocrine disorders | Metabolic |
| E702 | Disorders of tyrosine metabolism | Metabolic |
| E71 | Disorders of branched-chain amino-acid metabolism and fatty-acid metabolism | Metabolic |
| E72 | Other disorders of amino-acid metabolism | Metabolic |
| E74 | Other disorders of carbohydrate metabolism | Metabolic |
| E75 | Disorders of sphingolipid metabolism and other lipid storage disorders | Metabolic |
| E76 | Disorders of glycosaminoglycan metabolism | Metabolic |
| E77 | Disorders of glycoprotein metabolism | Metabolic |
| E791 | Lesch-Nyhan syndrome | Metabolic |
| E830 | Disorders of copper metabolism | Metabolic |
| E84 | Cystic fibrosis | Respiratory |
| E880 | Disorders of plasma-protein metabolism, not elsewhere classified | Metabolic |
| E881 | Lipodystrophy, not elsewhere classified | Metabolic |
| F803 | Acquired aphasia with epilepsy [Landau-Kleffner] | Neurology |
| F842 | Rett's syndrome | Neurology |
| G10 | Huntington's disease | Neurology |
| G111 | Early-onset cerebellar ataxia | Neurology |
| G113 | Cerebellar ataxia with defective DNA repair | Neurology |
| G12 | Spinal muscular atrophy and related syndromes | Neurology |
| G20 | Parkinson's disease | Neurology |
| G230 | Hallervorden-Spatz disease | Neurology |
| G238 | Other specified degenerative diseases of basal ganglia | Neurology |
| G318 | Other specified degenerative diseases of nervous system | Neurology |
| G319 | Degenerative disease of nervous system, unspecified | Neurology |
| G35 | Multiple sclerosis | Neurology |

**eTable 1. Life-limiting condition ICD-10 codes (cont)**

| **All** | **ICD-10 Description** | **Category** |
| --- | --- | --- |
| G404 | Other generalized epilepsy and epileptic syndromes | Neurology |
| G405 | Special epileptic syndromes | Neurology |
| G600 | Hereditary motor and sensory neuropathy | Neurology |
| G601 | Refsum's disease | Neurology |
| G702 | Congenital and developmental myasthenia | Neurology |
| G709 | Myoneural disorder, unspecified | Neurology |
| G710 | Muscular dystrophy | Neurology |
| G711 | Myotonic disorders | Neurology |
| G712 | Congenital myopathies | Neurology |
| G713 | Mitochondrial myopathy, not elsewhere classified | Neurology |
| G800 | Spastic quadriplegic cerebral palsy | Neurology |
| G808 | Other cerebral palsy | Neurology |
| G823 | Flaccid tetraplegia | Neurology |
| G824 | Spastic tetraplegia | Neurology |
| G825 | Tetraplegia, unspecified | Neurology |
| G934 | Encephalopathy, unspecified | Neurology |
| G936 | Cerebral edema | Neurology |
| G937 | Reye's syndrome | Neurology |
| H111 | Conjunctival degenerations and deposits | Other |
| H355 | Hereditary retinal dystrophy | Other |
| H498 | Other paralytic strabismus | Other |
| I21 | Acute myocardial infarction | Circulatory |
| I270 | Primary pulmonary hypertension | Circulatory |
| I42 | Cardiomyopathy | Circulatory |
| I613 | Nontraumatic intracerebral hemorrhage in brain stem | Circulatory |
| I81 | Portal vein thrombosis | Circulatory |
| J841 | Other interstitial pulmonary diseases with fibrosis | Respiratory |
| J96 | Respiratory failure, not elsewhere classified | Respiratory |
| J984 | Other disorders of lung | Respiratory |
| K550 | Acute vascular disorders of intestine | Gastrointestinal |
| K559 | Vascular disorder of intestine, unspecified | Gastrointestinal |
| K72 | Hepatic failure, not elsewhere classified | Gastrointestinal |
| K74 | Fibrosis and cirrhosis of liver | Gastrointestinal |
| K765 | Hepatic veno-occlusive disease | Gastrointestinal |
| K868 | Other specified diseases of pancreas | Gastrointestinal |
| M313 | Wegener granulomatosis | Other |
| M321 | Systemic lupus erythematosus with organ or system involvement | Other |
| M895 | Osteolysis | Other |
| N17 | Acute renal failure | Genitourinary |
| N18 | Chronic kidney disease | Genitourinary |
| N19 | Unspecified kidney failure | Genitourinary |
| N258 | Other disorders resulting from impaired renal tubular function | Genitourinary |
| P101 | Cerebral hemorrhage due to birth injury | Perinatal |
| P112 | Unspecified brain damage due to birth injury | Perinatal |
| P210 | Severe birth asphyxia | Perinatal |

**eTable 1. Life-limiting condition ICD-10 codes (cont)**

| **All** | **ICD-10 Description** | **Category** |
| --- | --- | --- |
| P285 | Respiratory failure of newborn | Perinatal |
| P290 | Neonatal cardiac failure | Perinatal |
| P293 | Persistent fetal circulation | Perinatal |
| P350 | Congenital rubella syndrome | Perinatal |
| P351 | Congenital cytomegalovirus infection | Perinatal |
| P358 | Other congenital viral diseases | Perinatal |
| P371 | Congenital toxoplasmosis | Perinatal |
| P524 | Intracerebral (nontraumatic) hemorrhage of newborn | Perinatal |
| P525 | Subarachnoid (nontraumatic) hemorrhage of newborn | Perinatal |
| P529 | Intracranial (nontraumatic) hemorrhage of newborn, unspecified | Perinatal |
| P832 | Hydrops fetalis not due to hemolytic disease | Perinatal |
| P912 | Neonatal cerebral leukomalacia | Perinatal |
| P916 | Hypoxic ischaemic encephalopathy of newborn | Perinatal |
| P960 | Congenital renal failure | Perinatal |
| Q000 | Anencephaly | Congenital |
| Q01 | Encephalocele | Congenital |
| Q031 | Atresia of foramina of Magendie and Luschka | Congenital |
| Q039 | Congenital hydrocephalus, unspecified | Congenital |
| Q040 | Congenital malformations of corpus callosum | Congenital |
| Q042 | Holoprosencephaly | Congenital |
| Q043 | Other reduction deformities of brain | Congenital |
| Q044 | Septo-optic dysplasia of brain | Congenital |
| Q046 | Congenital cerebral cysts | Congenital |
| Q049 | Congenital malformation of brain, unspecified | Congenital |
| Q070 | Arnold-Chiari syndrome | Congenital |
| Q200 | Common arterial trunk | Congenital |
| Q203 | Discordant ventriculoarterial connection | Congenital |
| Q204 | Double inlet ventricle | Congenital |
| Q206 | Isomerism of atrial appendages | Congenital |
| Q208 | Other congenital malformations of cardiac chambers and connections | Congenital |
| Q213 | Tetralogy of Fallot | Congenital |
| Q218 | Other congenital malformations of cardiac septa | Congenital |
| Q220 | Pulmonary valve atresia | Congenital |
| Q221 | Congenital pulmonary valve stenosis | Congenital |
| Q224 | Congenital tricuspid stenosis | Congenital |
| Q225 | Ebstein's anomaly | Congenital |
| Q226 | Hypoplastic right heart syndrome | Congenital |
| Q230 | Congenital stenosis of aortic valve | Congenital |
| Q232 | Congenital mitral stenosis | Congenital |
| Q234 | Hypoplastic left heart syndrome | Congenital |
| Q239 | Congenital malformation of aortic and mitral valves, unspecified | Congenital |
| Q254 | Other congenital malformations of aorta | Congenital |
| Q256 | Stenosis of pulmonary artery | Congenital |
| Q262 | Total anomalous pulmonary venous connection | Congenital |
| Q264 | Anomalous pulmonary venous connection, unspecified | Congenital |

**eTable 1. Life-limiting condition ICD-10 codes (cont)**

| **All** | **ICD-10 Description** | **Category** |
| --- | --- | --- |
| Q268 | Other congenital malformations of great veins | Congenital |
| Q282 | Arteriovenous malformation of cerebral vessels | Congenital |
| Q321 | Other congenital malformations of trachea | Congenital |
| Q336 | Congenital hypoplasia and dysplasia of lung | Congenital |
| Q396 | Congenital diverticulum of esophagus | Congenital |
| Q410 | Congenital absence, atresia and stenosis of duodenum | Congenital |
| Q419 | Congenital absence, atresia and stenosis of small intestine, part unspecified | Congenital |
| Q437 | Persistent cloaca | Congenital |
| Q442 | Atresia of bile ducts | Congenital |
| Q445 | Other congenital malformations of bile ducts | Congenital |
| Q447 | Other congenital malformations of liver | Congenital |
| Q601 | Renal agenesis, bilateral | Congenital |
| Q606 | Potter's syndrome | Congenital |
| Q614 | Renal dysplasia | Congenital |
| Q619 | Cystic kidney disease, unspecified | Congenital |
| Q642 | Congenital posterior urethral valves | Congenital |
| Q743 | Arthrogryposis multiplex congenita | Congenital |
| Q748 | Other specified congenital malformations of limb(s) | Congenital |
| Q750 | Craniosynostosis | Congenital |
| Q772 | Short rib syndrome | Congenital |
| Q773 | Chondrodysplasia punctata | Congenital |
| Q774 | Achondroplasia | Congenital |
| Q780 | Osteogenesis imperfecta | Congenital |
| Q785 | Metaphyseal dysplasia | Congenital |
| Q792 | Exomphalos | Congenital |
| Q793 | Gastroschisis | Congenital |
| Q804 | Harlequin fetus | Congenital |
| Q81 | Epidermolysis bullosa | Congenital |
| Q821 | Xeroderma pigmentosum | Congenital |
| Q824 | Ectodermal dysplasia (anhidrotic) | Congenital |
| Q858 | Other phakomatoses, not elsewhere classified | Congenital |
| Q860 | Fetal alcohol syndrome (dysmorphic) | Congenital |
| Q870 | Congenital malformation syndromes predominantly affecting facial appearance | Congenital |
| Q871 | Congenital malformation syndromes predominantly associated with short stature | Congenital |
| Q872 | Congenital malformation syndromes predominantly involving limbs | Congenital |
| Q878 | Other specified congenital malformation syndromes, not elsewhere classified | Congenital |
| Q91 | Edwards syndrome and Patau syndrome | Congenital |
| Q920 | Whole chromosome trisomy, nonmosaicism (meiotic nondisjunction) | Congenital |
| Q921 | Whole chromosome trisomy, mosaicism (mitotic nondisjunction) | Congenital |
| Q924 | Duplications seen only at prometaphase | Congenital |
| Q927 | Triploidy and polyploidy | Congenital |
| Q932 | Chromosome replaced with ring, dicentric or isochromosome | Congenital |
| Q933 | Deletion of short arm of chromosome 4 | Congenital |
| Q934 | Deletion of short arm of chromosome 5 | Congenital |

**eTable 1. Life-limiting condition ICD-10 codes (cont)**

| **All** | **ICD-10 Description** | **Category** |
| --- | --- | --- |
| Q935 | Other deletions of part of a chromosome | Congenital |
| Q938 | Other deletions from the autosomes | Congenital |
| Q952 | Balanced autosomal rearrangement in abnormal individual | Congenital |
| T860 | Bone-marrow transplant rejection | Other |
| T862 | Heart transplant failure and rejection | Other |
| Z515 | Encounter for palliative care | Other |
