# Supplementary material for: Deaths in children in England from SARS-CoV-2 infection during the first 2 years of the pandemic: a cohort study
Source: BMJ Open. 2025 Feb 5;15(2):e092627. doi: 10.1136/bmjopen-2024-092627 (PMC11800287; doi:10.1136/bmjopen-2024-092627)
Supplement: online supplemental file 3 [file bmjopen-15-2-s003.docx]

**eTable 3. ICD-10 codes for specific conditions**

| **Condition or Group** | **ICD-10 codes** |
| --- | --- |
| Asthma | J45.0, J45.8, J45.9 |
| Diabetes | E10-E14, G59.0, G63.2, I79.2, M14.2, N08.3, O24, Y42.3 |
| Epilepsy | F80.3, G40.0-G40.4, G40.6-G40.9, G41, R56.8, Y46.0-Y46.6 |
| Sickle Cell | D57.0, D57.1 |
| Trisomy 21 | Q90.0, Q90.1, Q90.2, Q90.9 |
| Oncology | C00-C97, D33, D43, D44.4, D48 |
| Congenital heart disease | Q20-Q26, Q89.3 |
| Cystic Fibrosis | E84, P75 |
| Life-limiting Neurodisability | A17, A81.0, F80.3 ,F84.2, G10 ,G11.1, G11.3, G12, G20, G23.0, G23.8, G31.8, G31.9, G35, G40.4, G40.5, G60.0, G60.1, G70.2, G70.9, G71.0, G71.1 ,G71.2, G71.3, G80.0, G80.8 ,G82.3, G82.4, G82.5 ,G93.4 ,G93.6, G93.7 |
| Preterm Birth | P07 or CDOP recording of birth before 37 weeks gestation. |
