# Supplementary material for: Deaths in children in England from SARS-CoV-2 infection during the first 2 years of the pandemic: a cohort study
Source: BMJ Open. 2025 Feb 5;15(2):e092627. doi: 10.1136/bmjopen-2024-092627 (PMC11800287; doi:10.1136/bmjopen-2024-092627)
Supplement: online supplemental file 4 [file bmjopen-15-2-s004.docx]

**eTable 4. Numbers of death associated, linked, and caused by COVID-19; split by child characteristics.**

| **Characteristic** | **Died of other causes** | | | | **Died of COVID-19** | |  |
| --- | --- | --- | --- | --- | --- | --- | --- |
|  | **Died without positive test for SARS-CoV-2** | | **Incidental positive SARS-CoV-2 test at death** | |  |  |  |
|  | **n** | **%** | **n** | **%** | **n** | **%** | **P^a^** |
| All Deaths | 6043 | 94.6% | 258 | 4.0% | 88 | 1.4% | - |
| Age |  |  |  |  |  |  | <.001 |
| <28 days | 2493 | 99.5% | 6 | 0.2% | 6 | 0.2% |  |
| 28-365 days | 1322 | 96.2% | 41 | 3.0% | 11 | 0.8% |  |
| 1-2 years | 245 | 90.4% | 20 | 7.4% | 6 | 2.2% |  |
| 2-4 years | 377 | 91.7% | 27 | 6.6% | 7 | 1.7% |  |
| 5-9 years | 410 | 89.5% | 35 | 7.6% | 13 | 2.8% |  |
| 10-14 years | 532 | 87.5% | 56 | 9.2% | 21 | 3.5% |  |
| 15-17 years | 664 | 87.3% | 73 | 9.6% | 24 | 3.2% |  |
| Sex |  |  |  |  |  |  | .30 |
| Female | 2603 | 94.2% | 116 | 4.2% | 43 | 1.6% |  |
| Male | 3411 | 94.8% | 142 | 4.0% | 45 | 1.3% |  |
| Ethnicity |  |  |  |  |  |  | .001 |
| Asian or Asian British | 994 | 93.3% | 44 | 4.1% | 28 | 2.6% |  |
| Black or Black British | 463 | 93.5% | 21 | 4.2% | 11 | 2.2% |  |
| Mixed | 361 | 95.0% | 14 | 3.7% | 5 | 1.3% |  |
| Other | 143 | 93.5% | 8 | 5.2% | 2 | 1.3% |  |
| White | 3793 | 94.8% | 168 | 4.2% | 41 | 1.0% |  |
| Deprivation |  |  |  |  |  |  | .01 |
| 1/2 | 2014 | 93.8% | 98 | 4.6% | 36 | 1.7% |  |
| 3/4 | 1366 | 95.3% | 52 | 3.6% | 15 | 1.1% |  |
| 5/6 | 1051 | 94.6% | 36 | 3.2% | 24 | 2.2% |  |
| 7/8 | 852 | 95.4% | 36 | 4.0% | 5 | 0.6% |  |
| 9/10 | 728 | 94.3% | 36 | 4.7% | 8 | 1.0% |  |
| Region |  |  |  |  |  |  | .75 |
| East Midlands | 498 | 95.0% | 21 | 4.0% | 5 | 1.0% |  |
| East of England | 569 | 95.0% | 22 | 3.7% | 8 | 1.3% |  |
| London | 1021 | 94.0% | 47 | 4.3% | 18 | 1.3% |  |
| North East | 301 | 95.6% | 11 | 3.5% | 3 | 1.0% |  |
| North West | 897 | 94.0% | 44 | 4.6% | 14 | 1.5% |  |
| South East | 806 | 93.9% | 40 | 4.7% | 14 | 1.5% |  |
| South West | 459 | 95.0% | 17 | 3.5% | 7 | 1.5% |  |
| West Midlands | 819 | 95.2% | 34 | 4.0% | 7 | 0.8% |  |
| Yorkshire and Humber | 673 | 95.6% | 22 | 3.1% | 13 | 1.4% |  |
| Vaccination Status (Child) |  |  |  |  |  |  | - |
| Fully Vaccinated | 72 | 85.7% | 12 | 14.3% | 0 | 0.0% |  |
| Not Fully Vaccinated | 5971 | 94.7% | 246 | 3.9% | 88 | 1.4% |  |
| Vaccination Status (Mother) (Deaths <1 year of age) |  |  |  |  |  |  | - |
| Fully Vaccinated | 280 | 99.6% | 1 | 0.4% | 0 | 0.0% |  |
| Not Fully Vaccinated | 3535 | 98.3% | 46 | 1.3% | 17 | 0.5% |  |
| Place of Death |  |  |  |  |  |  | .23 |
| Home | 1036 | 92.8% | 71 | 6.4% | 10 | 0.9% |  |
| Hospice | 239 | 90.2% | 24 | 9.1% | 2 | 0.8% |  |
| Hospital | 4398 | 95.6% | 133 | 2.9% | 72 | 1.6% |  |
| Abroad/Other/Unknown | 370 | 91.6% | 30 | 7.4% | 4 | 1.0% |  |
| Learning Disability  (Deaths over 5-17 years of age) |  |  |  |  |  |  | .001 |
| No | 893 | 89.5% | 83 | 8.3% | 22 | 2.2% |  |
| Yes | 472 | 85.8% | 48 | 8.7% | 30 | 5.5% |  |

^a^ Chi^2^ comparison of frequencies seen between CYP likely dying of SAR-CoV-2 and all other children
