# Supplementary material for: Deaths in children in England from SARS-CoV-2 infection during the first 2 years of the pandemic: a cohort study
Source: BMJ Open. 2025 Feb 5;15(2):e092627. doi: 10.1136/bmjopen-2024-092627 (PMC11800287; doi:10.1136/bmjopen-2024-092627)
Supplement: online supplemental file 5 [file bmjopen-15-2-s005.docx]

**eTable 5. Numbers of death associated, linked, and caused by COVID-19; split by life Limiting and Chronic Conditions (n=5444)**

| **Characteristic** |  | **Died of other causes** | | | | **Died of COVID-19** | |
| --- | --- | --- | --- | --- | --- | --- | --- |
|  |  | **Died without positive test for SARS-CoV-2** | | **Incidental positive SARS-CoV-2 test at death** | |  | |
|  |  | **n** | **%** | **N** | **%** | **n** | **%** |
| Number of LLC conditions | 0 | 1877 | 96.7% | 48 | 2.5% | 16 | 0.8% |
|  | 1 | 1474 | 97.0% | 31 | 2.0% | 14 | 0.9% |
|  | 2 | 861 | 93.3% | 45 | 4.9% | 17 | 1.8% |
|  | 3+ | 947 | 89.3% | 84 | 7.9% | 30 | 2.8% |
|  |  |  |  |  |  |  |  |
| Number of Chronic conditions | 0 | 1215 | 97.7% | 20 | 1.6% | 9 | 0.7% |
|  | 1 | 655 | 98.1% | 10 | 1.5% | 3 | 0.5% |
|  | 2 | 717 | 97.8% | 10 | 1.4% | 6 | 0.8% |
|  | 3+ | 2572 | 91.9% | 168 | 6.0% | 59 | 2.1% |
|  |  |  |  |  |  |  |  |
| LLC Categories |  |  |  |  |  |  |  |
| Neurological |  | 991 | 89.7% | 80 | 7.2% | 34 | 3.1% |
| Haematology |  | 401 | 88.1% | 49 | 10.8% | 5 | 1.1% |
| Oncology |  | 482 | 90.1% | 50 | 9.4% | 3 | 0.6% |
| Metabolic |  | 244 | 90.0% | 19 | 7.0% | 8 | 3.0% |
| Respiratory |  | 484 | 87.8% | 34 | 6.2% | 33 | 6.0% |
| Circulatory |  | 172 | 90.5% | 10 | 5.3% | 8 | 4.2% |
| Gastrointestinal |  | 247 | 89.8% | 21 | 7.6% | 7 | 2.6% |
| Genitourinary |  | 635 | 89.1% | 54 | 7.6% | 24 | 3.4% |
| Perinatal |  | 1382 | 97.4% | 24 | 1.7% | 13 | 0.9% |
| Congenital |  | 1142 | 94.5% | 49 | 4.1% | 17 | 1.4% |
| Other |  | 618 | 90.6% | 53 | 7.8% | 11 | 1.6% |
