# Supplementary material for: Deaths in children in England from SARS-CoV-2 infection during the first 2 years of the pandemic: a cohort study
Source: BMJ Open. 2025 Feb 5;15(2):e092627. doi: 10.1136/bmjopen-2024-092627 (PMC11800287; doi:10.1136/bmjopen-2024-092627)
Supplement: online supplemental file 6 [file bmjopen-15-2-s006.docx]

**eTable 6. Numbers of death associated, linked, and caused by COVID-19; split by Specific Chronic and LLC Conditions (n=5444)**

| **Characteristic** |  | **Died of other causes** | | | | **Died of COVID-19** | |
| --- | --- | --- | --- | --- | --- | --- | --- |
|  |  | **Died without positive test for SARS-CoV-2** | | **Incidental positive SARS-CoV-2 test at death** | |  |  |
|  |  | **n** | **%** | **n** | **%** | **n** | **%** |
| Specific Additional Conditions |  |  |  |  |  |  |  |
| Asthma |  | 158 | 97.8% | 17 | 9.4% | 5 | 2.8% |
| Diabetes |  | 39 | 97.5% | 1 | 2.5% | 0 | 0.0% |
| Epilepsy |  | 742 | 88.9% | 66 | 7.9% | 27 | 3.2% |
| Sickle cell disease |  | 3 | 75.0% | 0 | 0.0% | 1 | 25.0% |
| Trisomy 21 |  | 60 | 82.2% | 7 | 9.6% | 6 | 8.2% |
| Oncology |  | 482 | 90.1% | 50 | 9.4% | 3 | 0.6% |
| Cardiology (Congenital) |  | 785 | 92.7% | 43 | 5.1% | 19 | 2.2% |
| Life-limiting Neurodisability |  | 991 | 89.7% | 80 | 7.2% | 34 | 3.1% |
| Cystic Fibrosis |  | 6 | 75.0% | 0 | 0.0% | 2 | 25.0% |
| Chronic Renal Failure |  | 50 | 83.3% | 8 | 13.3% | 2 | 3.3% |
| Cerebral Palsy |  | 343 | 88.2% | 29 | 7.5% | 17 | 4.4% |
| Preterm Birth |  | 2399 | 97.5% | 40 | 1.6% | 22 | 0.9% |
