# Supplementary material for: Deaths in children in England from SARS-CoV-2 infection during the first 2 years of the pandemic: a cohort study
Source: BMJ Open. 2025 Feb 5;15(2):e092627. doi: 10.1136/bmjopen-2024-092627 (PMC11800287; doi:10.1136/bmjopen-2024-092627)
Supplement: Supplementary file 7 [file bmjopen-2024-092627.reviewer_comments.pdf]

# PEER REVIEW HISTORY

BMJ Open publishes all reviews undertaken for accepted manuscripts. Reviewers are asked to complete a checklist review form and are provided with free text boxes to elaborate on their assessment. These free text comments are reproduced below.

**ARTICLE DETAILS**

**Title (Provisional)**

Deaths in Children in England from SARS-CoV-2 infection during the first two years of the pandemic: A cohort study

**Authors**

Odd, David; Stoianova, Sylvia; Williams, Tom; Thursby-Pelham, Anna; Ladhani, Shamez N; Oligbu, Godwin; Fleming, Peter; Luyt, Karen

---

**VERSION 1 - REVIEW**

---

| | |
|---|---|
| **Reviewer** | **1** |
| **Name** | **Pierce, Matthias** |
| **Affiliation** | **University of Manchester** |
| **Date** | **14-Oct-2024** |
| **COI** | **None** |

---

CYP needs defining

The study provides an overview of the epidemiology of young people who died during the COVID-19 pandemic who had as COVID-19 as their likely cause of death. The method for identifying COVID-19 deaths appears methodologically sound. However, the statistical analyses are not well described - it is not clear what the denominator for the analysis is: is this the general population? Or those with a positive test? Why are you comparing deaths from COVID with deaths from other causes?

It is not clear how LLC were handled in the analysis: is this a separate cohort? How was time considered in this analysis?

---

| | |
|---|---|
| **Reviewer** | **2** |
| **Name** | **Dzurova, Dagmar** |
| **Affiliation** | **Charles University, Department of Social Geography and Regional Development** |

| | |
|---|---|
| **Date** | **25-Nov-2024** |
| **COI** | **None** |

---

I thank you for the opportunity to review this study. I was very interested in the topic, and there is a great need for research on child health in the context of the impact of the COVID-19 pandemic. This is especially so when a quality data source is available, as in this evaluated study—the NCMD data source, which allows for unique analyses of COVID-19-Related deaths.

However, I regret to say that the RESULTS section is unsuitable for publication in the presented form. The resulting analyses are not well described, and I traced a number of inconsistencies, see below for details. I recommend that more attention be paid to the RESULTS section in particular and that the text be largely edited.

I hereby apologize to the authors of the reviewed article for my linguistic imperfection, but English is not my first language. Nevertheless, I have tried to describe as many recommendations as possible to improve the text.

Page 3, line 30

I recommend checking here: (< 5 years; 4.68 [.16-6.68]), according to Table 1 the interval should be [3.16-6.68]

Page 7, Line 6-15

In this part of the text, the connection to the subsequent analysis escapes me. Please provide a clearer description.

Page 10, Line 23

Link to eTable 6 - Table not found

Page 19, Table 1

The "p" value in the Table 1 is better adjusted to a uniform format, preferably 3 decimal places.

There is one less case for the Ethnicity variable (not 88 deaths, but only 87) - error or other reason?

Page 21, Table 2

The title of Table 2 does not match the content of the Table, here are the results of a simple multivariate logistic regression.

The number of 78 COVID-19 deaths is given in the text, but Table 2 shows 77 COVID-19 deaths. How do you explain this?

I recommend that the explanation under the table "dying of SAR-CoV-2" be merged with "dying of COVID-19".

Page 22, Table 3

Isn't the number of deaths misstated in the table title? I believe it should not be 5333 but 5444 (5367+77).

Aren't the * marks explained what they mean? Conversely, the explanatory marks "a, b" in case a do not have a reference

It is not clear to me the numbers (superscripts) given in the last column, e.g. for Asthma 2,000,027 11. Is this a misrepresentation? Please check.

Page 23, Figure 1

Chart type: The number of deaths for each month represents separate values associated with specific time points. However, a line chart gives the impression of a continuous connection between these points, which may not be consistent with the nature of the data. although a line chart highlights trends, I would recommend a bar chart, which better matches the nature of the data and avoids potential ambiguity in interpretation.

Interpretation: The figure is inadequately interpreted in one sentence, with no explanation of the two variables shown—likely COVID-19 deaths and Possible COVID deaths.

Content: Would it not be more beneficial to show the proportion of COVID-19 deaths out of all deaths?

Location of Figure 1: I recommend including Figure 1 in the introduction of the results, not in the sub-section on mortality rates.

Page 24, Figure 2

The title of the figure is not apt given the content of the Table.

The description referring to Figure 2 and Table 1 is not entirely correct, as the table has age categories, but the figure has age units.

---

## VERSION 1 - AUTHOR RESPONSE

**Reviewer: 1**
**Mr. Matthias Pierce, University of Manchester**

Comments to the Author:
CYP needs defining

* Amended

The study provides an overview of the epidemiology of young people who died during

the COVID-19 pandemic who had as COVID-19 as their likely cause of death. The method for identifying COVID-19 deaths appears methodologically sound. However, the statistical analyses are not well described - it is not clear what the denominator for the analysis is: is this the general population? Or those with a positive test? Why are you comparing deaths from COVID with deaths from other causes?
It is not clear how LLC were handled in the analysis: is this a separate cohort? How was time considered in this analysis?
* Thank you for reviewing the paper, and we have expanded the manuscript to make a number of things clearer after these recommendations. In particular:

Comment: Denominator for the analysis is: is this the general population? Or those with a positive test?

* Rates were calculated for the whole population, and we have expanded the methods around this.

Why are you comparing deaths from COVID with deaths from other causes?
* The NCMD data is limited to children who have died, and much of the data (esp. robust COVID-19) testing was only available in these children: and hence cannot derived case fatality rates for COVID-19 in this work. However, we did have population numbers, from ONS, and other sources, for many characteristics, allowing us to place the risk of dying in context. We have expanded the discussion to reflect this.

It is not clear how LLC were handled in the analysis: is this a separate cohort?

* We have expanded the methods around this, which we hope is now clearer.

Comment: For the LLC analysis, time was not used as factors. How was time considered in this analysis?
* Apart from the specific graph showing number of deaths by date, we did not investigate if the relationships we identified varied by time, and due to the small numbers, we feel this would be challenging to interpret. We have how expanded on the discussion around Figure 1 as part of this review process.

**Reviewer: 2**
**Dr. Dagmar Dzurova, Charles University**

Page 3, line 30: I recommend checking here: (< 5 years; 4.68 [.16-6.68]), according to

Table 1 the interval should be [3.16-6.68]
* Corrected to [3.16-6.68]

Page 7, Line 6-15
In this part of the text, the connection to the subsequent analysis escapes me. Please provide a clearer description.
* We wanted to be clear how this data may differ from the data we published before, as some overlap exists. We have expanded this section with that in mind.

Page 10, Line 23
Link to eTable 6 - Table not found
* Apologies: this failed to be uploaded. We have added it.

Page 19, Table 1
The "p" value in the Table 1 is better adjusted to a uniform format, preferably 3 decimal places.
* We are happy to amend the format of the p-values presented as per editorial preference.

There is one less case for the Ethnicity variable (not 88 deaths, but only 87) - error or other reason?
* One infant had missing data on ethnicity. We have added a footnote to make this clear.

Page 21, Table 2
The title of Table 2 does not match the content of the Table, here are the results of a simple multivariate logistic regression.

* The table shows the number, proportion and relative odds of both Chronic and Life Limiting conditions in those children you did, or did not die of COVID-19. We have expanded the title to make this clearer.

The number of 78 COVID-19 deaths is given in the text, but Table 2 shows 77 COVID-19 deaths. How do you explain this?
* The '78' is an error, and has been amended. All other number and percentages have been re-checked and are correct.

I recommend that the explanation under the table "dying of SAR-CoV-2" be merged with "dying of COVID-19".
* Amended as suggested

Page 22, Table 3
Isn't the number of deaths misstated in the table title? I believe it should not be 5333 but 5444 (5367+77).

* Amended

Aren't the * marks explained what they mean? Conversely, the explanatory marks "a, b" in case a do not have a reference
* The '*' should refer to the 'a' footnote (amended). The 'b' footnote relates to the OR for DM, which is a one-sided test. We have expanded the explanation to make sure it is clearer.

It is not clear to me the numbers (superscripts) given in the last column, e.g. for Asthma 2,000,027 11. Is this a misrepresentation? Please check.
* The numbers are the references for the estimated population at risk. To make the paper clearer we have removed them (they are present in the text).

Page 23, Figure 1: Chart type: The number of deaths for each month represents separate values associated with specific time points. However, a line chart gives the impression of a continuous connection between these points, which may not be consistent with the nature of the data. although a line chart highlights trends, I would recommend a bar chart, which better matches the nature of the data and avoids potential ambiguity in interpretation.

* We used a line chart as the two data sources (Likely and Possible COVID-19 deaths) were very difficult to distinguish when plotted both as columns. We would be happy to change the chart-type on the editor's preference of course.

Interpretation: The figure is inadequately interpreted in one sentence, with no explanation of the two variables shown—likely COVID-19 deaths and Possible COVID deaths.

* We have expanded the description and interpretation as suggested.

Content: Would it not be more beneficial to show the proportion of COVID-19 deaths out of all deaths?
* Thank you for your comment, but we have published some separate work looking at the proportion of deaths across the pandemic; and this work was aimed as identify the characteristics of those who died.

Location of Figure 1: I recommend including Figure 1 in the introduction of the results, not in the sub-section on mortality rates.
* We have moved the reference/description as suggested.

Page 24, Figure 2: The title of the figure is not apt given the content of the Table. The description referring to Figure 2 and Table 1 is not entirely correct, as the table has age categories, but the figure has age units.
* We have re-titled the figure, which we hope is more descriptive and correct.
